# Interactions of *Francisella tularensis* with Alveolar Type II Epithelial Cells and the Murine Respiratory Epithelium

**DOI:** 10.1371/journal.pone.0127458

**Published:** 2015-05-26

**Authors:** Matthew Faron, Joshua R. Fletcher, Jed A. Rasmussen, Michael A. Apicella, Bradley D. Jones

**Affiliations:** 1 Graduate Program in Genetics, University of Iowa, Iowa City, Iowa, United States of America; 2 Department of Microbiology, University of Iowa, Iowa City, Iowa, United States of America; Université Paris Descartes, FRANCE

## Abstract

*Francisella tularensis* is classified as a Tier 1 select agent by the CDC due to its low infectious dose and the possibility that the organism can be used as a bioweapon. The low dose of infection suggests that *Francisella* is unusually efficient at evading host defenses. Although ~50 cfu are necessary to cause human respiratory infection, the early interactions of virulent *Francisella* with the lung environment are not well understood. To provide additional insights into these interactions during early *Francisella* infection of mice, we performed TEM analysis on mouse lungs infected with *F*. *tularensis* strains Schu S4, LVS and the O-antigen mutant Schu S4 *waaY*::TrgTn. For all three strains, the majority of the bacteria that we could detect were observed within alveolar type II epithelial cells at 16 hours post infection. Although there were no detectable differences in the amount of bacteria within an infected cell between the three strains, there was a significant increase in the amount of cellular debris observed in the air spaces of the lungs in the Schu S4 *waaY*::TrgTn mutant compared to either the Schu S4 or LVS strain. We also studied the interactions of *Francisella* strains with human AT-II cells *in vitro* by characterizing the ability of these three strains to invade and replicate within these cells. Gentamicin assay and confocal microscopy both confirmed that *F*. *tularensis* Schu S4 replicated robustly within these cells while *F*. *tularensis* LVS displayed significantly lower levels of growth over 24 hours, although the strain was able to enter these cells at about the same level as Schu S4 (1 organism per cell), as determined by confocal imaging. The Schu S4 *waaY*::TrgTn mutant that we have previously described as attenuated for growth in macrophages and mouse virulence displayed interesting properties as well. This mutant induced significant airway inflammation (cell debris) and had an attenuated growth phenotype in the human AT-II cells. These data extend our understanding of early *Francisella* infection by demonstrating that *Francisella* enter significant numbers of AT-II cells within the lung and that the capsule and LPS of wild type Schu S4 helps prevent murine lung damage during infection. Furthermore, our data identified that human AT-II cells allow growth of Schu S4, but these same cells supported poor growth of the attenuated LVS strain *in vitro*. Collectively, these data further our understanding of the role of AT-II cells in *Francisella* infections.

## Introduction

*Francisella tularensis* is a highly virulent intracellular bacterial pathogen that causes the human infectious disease tularemia [1, 2]. The most common route of infection is cutaneous, although infection via the respiratory route is highly efficient and can cause a lethal infection in 30–60% of patients that do not receive treatment [3]. In mice, respiratory infection with a single virulent *F*. *tularensis* organism is virtually always lethal while in a human as few as 50 organisms are believed to result in a potentially lethal infection [4, 5]. The ability to weaponize this organism for respiratory delivery, along with the low infective dose and the high lethality of *Francisella tularensis* are the reasons why this organism is classified as a Tier 1 select agent by the Centers for Disease Control and Prevention (CDC).

In an effort to understand early events in *Francisella* infection and how they can reproducibly lead to lethal respiratory disease, it was of interest to examine the interactions between *Francisella* and the alveolar air spaces. In general, the lung is protected from microbial insult by both alveolar macrophages that reside in the extracellular alveolar air spaces and by the physical barrier composed of alveolar epithelial cells. The alveolar macrophages are loosely associated with the epithelium, and are in a relatively inactivated state where they function to engulf particles that are inhaled during breathing [6]. Upon engulfment of a particle or bacterium, alveolar macrophages increase their phagocytic activity, oxidative burst capacity and production of pro-inflammatory cytokines [7]. These induced protective responses lead to the release of alveolar macrophages from the airway epithelium, where they (along with their engulfed cargo) are removed from the lung air spaces via the mucociliary escalator [8]. As an early line of defense in the lungs, these activities are designed to engage and direct bacteria away from the alveolar epithelium. Since interactions with alveolar macrophages are likely to result in the removal of *Francisella* organisms from airway epithelial environment, it seems likely that the bacteria must productively interact with other cell types in order to breach the respiratory epithelium and gain access to deeper tissue and the bloodstream.

Besides alveolar macrophages, the alveolus is composed of two other cell types: alveolar epithelial type I (AT-I) and alveolar epithelial type II cells (AT-II), which are important components of a physical barrier to protect deeper tissues from microbes and airborne particles. AT-I cells are thin, elongated cells that comprise 95% of the alveolus surface area and are important in maintaining the structure of the alveolus and facilitating gas exchange [9]. In contrast, AT-II cells are smaller spherical cells that contain microvilli and lamellar bodies [10, 11]. These cells constitute the remaining 5% of the epithelial surface, but represent 60% of the alveolar epithelial cells [12]. AT-II cells have diverse functions within the lung, and are involved in several processes, including: secretion of surfactant, regeneration of the alveolar epithelium, and protecting against bacterial invasion [13]. AT-II cells protect against pathogens by sensing pathogens through TLR stimulation [14, 15], secretion of anti-microbial peptides [16], and both activation and deactivation of inflammation through modulation of cytokines and chemokines [17]. However, it has been shown that pathogenic bacteria such as *Mycobacterium tuberculosis*, can survive these anti-microbial mechanisms and be internalized into AT-II cells, which allows the bacteria to cross the alveolar barrier and gain access to the bloodstream [18].

Several studies have provided evidence that airway epithelial cells may be an important access point for *Francisella* to initiate disease in the lung. Using mice intranasally infected with *F*. *tularensis* LVS, Hall *et al*. [19] demonstrated that GFP-labelled organisms co-localized with cells that produced pro-surfactant protein C (pSP-C), a marker for AT-II cells, and that bacterial counts increased rapidly in the lungs over time, suggesting that the organisms were multiplying within type II airway epithelial cells. Another study by Hall *et al*. examined the types and percentages of murine lung cells infected after inhalation of *F*. *tularensis* U112 (*novicida*), *F*. *tularensis* LVS, or *F*. *tularensis* Schu S4 [20]. It was observed that these *F*. *tularensis* strains infected a wide variety of different lung cell types that included alveolar macrophages, neutrophils, dendritic cells, monocytes and alveolar type II cells, although the percentage of cells infected varied from strain to strain [20]. We, and others, have studied the interactions of *F*. *tularensis* with various types of epithelial cells [19, 21, 22]. These efforts have demonstrated that *F*. *tularensis* LVS is taken up by epithelial tissue culture cells where the organisms are able to grow ~1000-fold over 24 hours, which represents a growth rate comparable to that observed *in vitro* in growth media. Additionally, Gentry *et al*. has reported that interactions of *F*. *tularensis* with primary human AT-II cells stimulate a NF-kB-dependent cytokine release from AT-II lung epithelial cells suggesting that these cells play important roles in recruitment of immune cells through the pulmonary endothelium [23].

## Materials and Methods

### Bacterial strains and growth conditions

*F*. *tularensis* LVS, *F*. *tularensis* Schu S4 and the *F*. *tularensis waaY*::TrgTn (*FTT1236*) mutant in the Schu S4 background were routinely cultured on modified Mueller-Hinton agar plates (Acumedia) or in Mueller-Hinton broth supplemented with 1% glucose (wt/vol) 0.025% ferric pyrophosphate and 2% IsoVitaleX [24]. In preparation for experiments, strains were grown in modified Mueller-Hinton broth with aeration at 37°C. The antibiotics kanamycin and/or spectinomycin were added to the growth media at a concentration of 25 μg per ml when necessary.

### Epithelial cell culture

Primary human alveolar type 2 cells (AT-II) that have been immortalized with SV40 were purchased from Applied Biological Materials, Inc. (Richmond, BC, Canada) and cultured in PriGrow III medium (ABM, Inc.) supplemented with 10% fetal bovine serum in collagen-coated flasks at 37°C in 5% CO_2_, as directed by the supplier. When a tissue culture flask approached 80–90% confluency, the cells were lifted by trypsin-EDTA treatment, washed and then either passaged to another collagen-coated T-75 flask, seeded into collagen-coated wells of a 24 well dish or seeded into wells containing collagen-coated coverslips. The trypsinized cells were spun down and resuspended in 2–3 ml of fresh tissue culture media to remove the trypsin-EDTA. Cells were quantitated with a hemacytometer and then 1 ml of cell suspension containing 3 x 10^5^ cells was seeded into the desired number of wells of a 24 well tissue culture plate the day before the cells were to be infected.

### Quantitative epithelial cell growth assays

Approximately 3 x 10^5^ AT-II cells were infected at a multiplicity of infection of 100:1 with mid to late log phase (OD_600_ 0.5–0.9) cultures of *F*. *tularensis* SchuS4, *F*. *tularensis* SchuS4 *waaY*::TrgTn, or *F*. *tularensis* LVS. After 4 hours, the infected AT-II cells were washed three times with PBS before adding fresh tissue culture media containing 50 μg per ml of gentamicin for 1 hour to kill extracellular bacteria. After gentamicin treatment, cells were washed three times with PBS to remove the gentamicin and fresh antibiotic-free media was added to each well for the remainder of the assay. AT-II cell monolayers were lysed at 4, 16 or 24 hours with 1% saponin followed by serial dilution to quantitate the number of internalized bacteria in each well. Dilutions of the original inoculum were also plated and percent uptake was determined for the 4 hour time point. Bacteria recovered at later time points were compared to the 4 hour time point to determine the level of growth for each strain.

### Immunofluorescence staining and microscopy

AT-II cells were seeded onto collagen-coated coverslips in 24 well dishes and infected according to the protocol described above. At 4, 16 or 24 hours post-infection the infected cells on the coverslips were fixed with 10% formalin for 30 minutes, permeabilized with acetone/methanol (1:1) for 15 minutes, washed with PBS and then stored in blocking buffer until immunostaining was performed. Immunostaining was performed using mouse anti-GFP monoclonal antibody diluted 1:10,000 (Sigma). Secondary antibodies were acquired from the University of Iowa Central Microscopy and used at a dilution of 1:500. Samples were viewed and images were captured with a Zeiss LSM 710 confocal microscope.

### Murine infections

BALB/c female mice, 6–8 weeks of age, were purchased from the National Cancer Institute (NCI). Each mouse was anesthetized by inhalation of isoflurane and then immediately infected intranasally with *F*. *tularensis* SchuS4, *F*. *tularensis* SchuS4 *waaY*::TrgTn or *F*. *tularensis* LVS in a volume of 50 μl. Since there are published estimates that mouse lungs contain approximately 10^6^ alveoli [25–27], approximately 10^8^ cfu of each strain (MOI of 100) were inoculated intranasally to significantly increase the probability of observing early interactions of the bacteria with lung cells. Bacterial doses were estimated from OD_600_ readings and were confirmed by serial dilution and enumeration on agar plates. All work with viable *F*. *tularensis* strains was performed within the Carver College of Medicine Biosafety Level 3 (BSL3) Core Facility and all experimental protocols were reviewed for safety by the BSL3 Oversight Committee of The University of Iowa Carver College of Medicine. Work with recombinant *F*. *tularensis* strains was approved by the Institutional Biosafety Committee at the University of Iowa. All animal experiments were performed according to procedures approved by The University of Iowa Institutional Animal Care and Use committee.

### Transmission electron microscopy

Mice were euthanized at either 16 or 24 hours post infection and lungs were perfused with 4% paraformaldehyde. Lungs were removed by dissection from each mouse and fixed in 4% paraformaldehyde for 2 days. After 2 days, one lung was homogenized and plated for a BSL-3 sterility control. The other lung samples were placed into 2.5% gluteraldehyde to await the sterility test result. Upon demonstration that samples were sterile, lung sections were prepared for transmission electron microscopy as previously described [28, 29]. Briefly, samples were washed 3 times in 0.2M sodium cacodylate buffer and then fixed in a 1% osmium, 1.5% potassium ferrocyanide solution for 1 hour. Samples were then washed for 15 minutes in a series of increasing ethanol concentrations starting with 50% ethanol, 75% ethanol, 95% ethanol, and ending with 100% ethanol. After the ethanol washes, Epon resin was perfused into each sample by equilibrating the samples for 1 hour each first in a 2:1 (ethanol:Epon) solution, then a 1:1 solution and finally a 1:2 solution. Samples were then placed in gel capsules and embedded in fresh Epon and placed in a 60°C oven overnight. 70 nm sections were cut from the hardened blocks using an ultramicrotome and the samples were counterstained with uranyl acetate and lead citrate. Sections were viewed on a JEM-1230 transmission electron microscope (Japan Electron Optics Laboratory Co., Peabody MA).

### Statistical analysis

Two-tailed Student’s T-tests were performed with two sample equal variance using Microsoft Excel software. Values of p < 0.01 were used as statistically significant.

## Results

### Growth patterns of virulent *F*. *tularensis* Schu S4, *waaY*::TrgTn and LVS in newly transformed human alveolar type 2 cells

**Fig 1:**
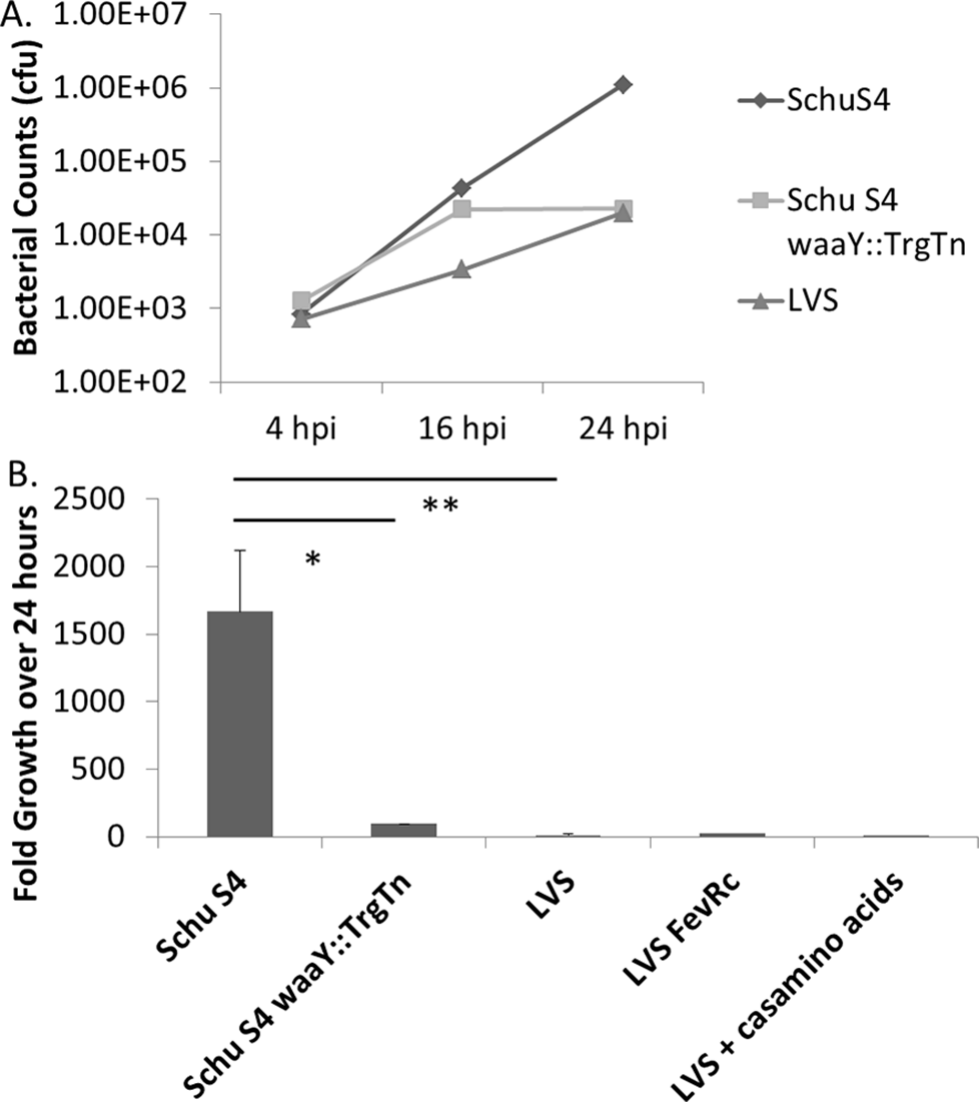
Growth of *F*. *tularensis* Schu S4, *F*. *tularensis waaY*::TrgTn and *F*. *tularensis* LVS in cultured human alveolar type II cells. Immortalized primary AT-II tissue culture cells were used to perform gentamicin protection assays. The ability of the three *Francisella* strains to enter and replicate with these epithelial lung cells was measured after infecting at an MOI 100:1. Fold growth was calculated as the difference between the number of bacteria surviving gentamicin treatment at 4 hours and 24 hours post-infection. (A) Data are a single experiment representative of three separate experiments. (B) Data are an average of four independent experiments.

Our research group and others have previously reported the entry and growth properties of *Francisella* strains within various tissue culture cell lines such as: A549s, HEp-2 cells, J774s, THP-1 cells, and others. [19, 21, 22, 30, 31]. While useful for dissecting aspects of intracellular growth, these cell lines have likely lost many of the characteristics of primary (or short passage) cells and thus may not accurately reflect key aspects of the host-pathogen interaction [32, 33]. Due to the variability of these cell lines, it is possible that important details about the growth of *F*. *tularensis* organisms in appropriate respiratory epithelial cells remain unknown. In order to better understand the interactions of *Francisella* with AT-II cells, we conducted experiments with recently immortalized primary human AT-II cells, to compare the interactions of virulent *F*. *tularensis* Schu S4, and the *F*. *tularensis* LVS strain in these cells. Additionally, we characterized the O-antigen mutant, Schu S4 *waaY*::TrgTn (FTT_1236), which has been previously reported to have 10-fold higher uptake and growth kinetics in human MDM cells [24]. The organism grows robustly within human MDMs until 16 hours post-infection when the organisms cease growing and it becomes difficult to detect cells on the slide to perform microscopy. The abilities of the three *Francisella* strains to enter and replicate within human AT-II cells were compared by infecting the cells with Schu S4, Schu S4 *waaY*::TrgTn, an attenuated LPS/capsule mutant, or LVS for either 4 or 24 hours. Both gentamicin protection assays and confocal microscopy were performed to acquire quantitative uptake and growth data, as well as to obtain qualitative data about the bacterial-host cell interactions. At 4 hours post-infection, the Schu S4 and LVS strains were detected entering into the primary human AT-II cells at comparable levels (Fig 1A). In comparison, the Schu *waaY*::TrgTn strain had uptake levels into the human AT-II cells that were consistently ~2-fold higher than that observed for either Schu S4 or LVS; however, this increase was not significant (Fig 1A). Interestingly, at the 24 hour time point there were significant differences in the bacterial burden in cells infected with Schu S4 compared to the cells infected with Schu S4 *waaY*::TrgTn or LVS (P < 0.05 and P < 0.01, respectively). Similar to work that our lab has previously reported for other tissue culture epithelial cell lines, Schu S4 entered and replicated ~1600-fold over the 24 hour course of the experiment (Fig 1B). We saw no apparent sign of cell death by cells infected with Schu S4 at 24 hours although we estimated that the cells that were infected contained 2,500–5,000 cfu. Conversely, following entry into human AT-II cells, the *F*. *tularensis* LVS strain grew significantly less well than wild type Schu S4, as we saw only 12-fold growth over 24 hours (Fig 1B).

One possibility to explain the difference in growth in the AT-II cells was a difference in FPI expression between *F*. *tularensis* Schu S4 and *F*. *tularensis* LVS. In previous work from our lab we demonstrated that Schu S4 expresses the FPI genes 3-fold higher than LVS [30]. To determine if the poor growth of LVS observed in the human AT-II cells might be due to low FPI expression, we increased FPI gene expression by overexpression of *fevR*. As a control, Miller assays were performed which demonstrated that overexpression of FevR from this plasmid increases an *iglA-lacZ* reporter (4-fold) (data not shown). However, even when *fevR* gene expression was substantially increased in LVS the AT-II cell growth defect was not rescued (Fig 1B). Additionally, it has been demonstrated that nutrient availability (i.e. amino acids) is important for *Francisella* cytosolic cell growth [34]. To determine if the poor growth of LVS is due to a lack of amino acids, Casamino acids (1% w/v) were added to the cell media and infections were performed. The addition of free amino acids to the growth medium of cells during infection did not rescue the LVS growth phenotype, however, since this was a negative result, it is possible that addition of Casamino acids did not increase cytosolic amino acid availability and so better controls are needed to definitively test if the LVS phenotype is due to low cytosolic amino acid concentrations (Fig 1B).

The growth pattern of the Schu S4 *waaY*::TrgTn strain in the human AT-II cells paralleled its growth pattern in human MDMs. Similar to MDM cells, there was significant growth within human AT-II cells until 16 hours post-infection [24], but then the bacteria failed to maintain that growth (Fig 1A). Since the Schu S4 *waaY*::TrgTn stops growing after ~16 hours within the human AT-II cells, a significant impact on the growth of the strain was observed (~90-fold growth over 24 hours) (Fig 1B).

**Fig 2:**
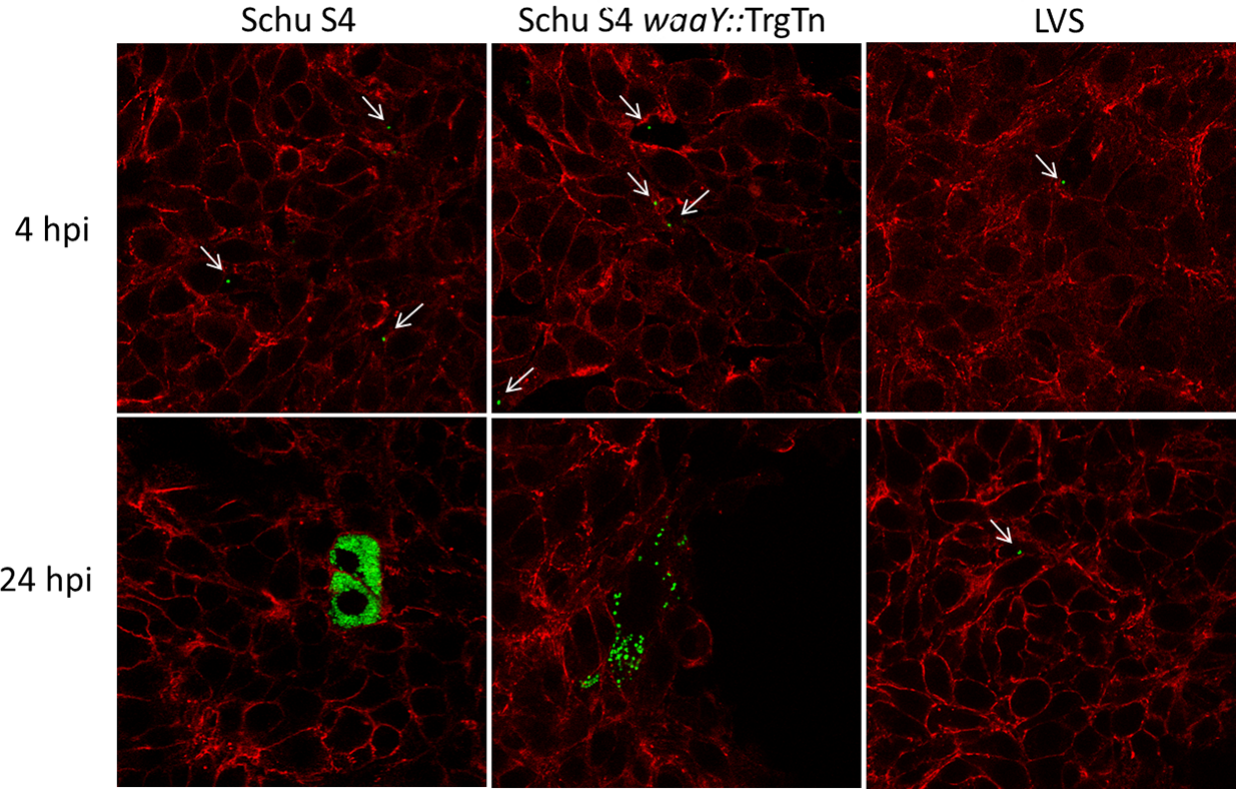
Confocal microscopy of *F*. *tularensis* Schu S4, *F*. *tularensis waaY*::TrgTn and *F*. *tularensis* LVS growth in cultured human alveolar type II cells. Immortalized primary AT-II cells were infected at an MOI of 100:1 and confocal microscopy was performed with cells stained for actin (red). In addition, each *Francisella* strain contained a plasmid encoding GFP and is represented as green. The thin white arrows point to single *Francisella* within cells.

As a second method to examine the interactions of *Francisella* strains with immortalized primary human AT-II cells, a similar set of experiments examined the interactions of the bacteria with these cells using confocal microscopy. As expected, there were no significant observable differences at 4 hours post-infection between Schu S4, Schu S4 *waaY*::TrgTn and the LVS strains (Fig 2). Cells containing bacteria typically had one organism per cell and no apparent replication had occurred at this time point (Fig 2). However, by 24 hours there were several notable differences between the three strains that correlated well with the bacterial count data shown in Fig 1. AT-II cells infected with *F*. *tularensis* Schu S4 clearly allowed vigorous replication as observed by the filling of cell cytosol with GFP-labeled *F*. *tularensis* (Fig 2). In contrast, the majority of LVS-infected cells at 24 hours post infection still contained only a single bacterium, suggesting that LVS was significantly impaired in its ability to replicate within the human AT-II cells compared to the Schu S4 strain (Fig 2). Some growth was observed within LVS infected cells, however, these cells were relatively rare and growth appeared to be limited to no more than a few rounds of replication. It is possible that these cells containing replicating LVS could have been damaged or less fit somehow, but this could not be ascertained by the confocal microscopy performed. Cells infected with *F*. *tularensis* Schu S4 *waaY*::TrgTn had an intermediate growth phenotype, in that infected cells contained a range of bacteria, from a couple of bacteria per cell, to cells nearly filled with organisms (Fig 2). The reduced growth phenotype is likely due to host cell recognition of the mutant, which lacks capsule and O-side chain additions to the lipid A-core sugar molecules in the outer membrane, and early cell death.

**Fig 3:**
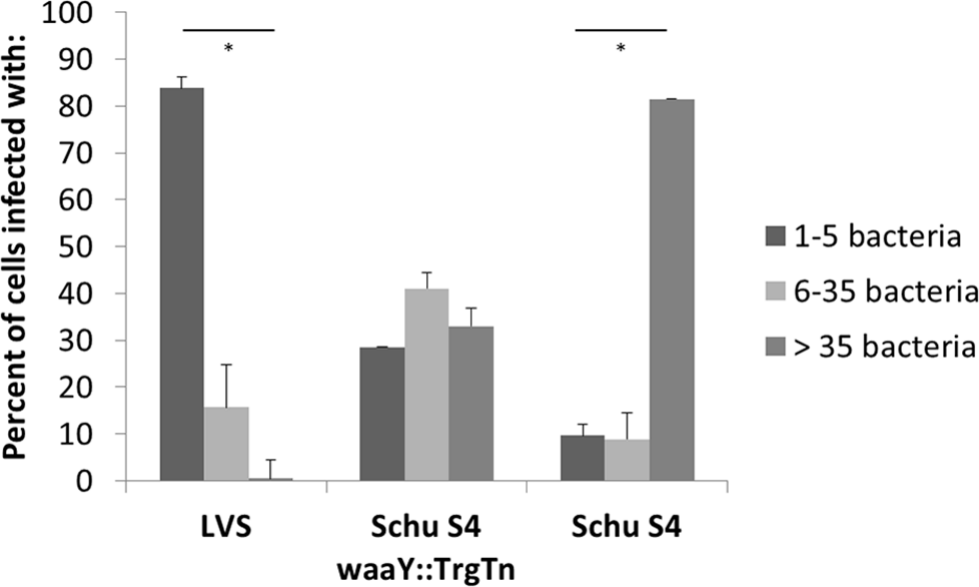
Scoring of growth patterns observed in confocal imaging. Infected cells were classified into three separate categories based on the amount of bacteria each cell contained at 24 hpi. Cells infected with Schu S4 had significantly more bacteria per cell compared to cells infected with LVS 24 hpi (*P <. 001). Percentages were calculated from greater than 300 cells containing organisms from three independent experiments.

To semi-quantitatively analyze the growth patterns of each of the three *Francisella* strains in AT-II cells, we scored infected cells for the number of bacteria that were inside an infected cell 24 hours post-infection. Cells were placed into one of three groups: 5 or less intracellular bacteria, between 6–35 intracellular bacteria, and greater than 35 intracellular bacteria. More than 300 cells for each strain were counted from three separate experiments. Using this scoring criteria, greater than 80% of the Schu S4-infected cells had robust growth with more than 35 bacteria, whereas there were almost no LVS-infected cells (<1%) that had greater than 35 bacteria (P < 0.001) (Fig 3). The majority (> 80%) of infected LVS cells had 1–5 bacteria per infected cell, while cells infected with the Schu S4 *waaY*::TrgTn strain separated into the three categories equally (Fig 3). These data provide a clearer picture that *F*. *tularensis* Schu S4 replicates robustly in human AT-II cells while *F*. *tularensis* LVS has a low capacity to grow in the cytosol of these cells, although it is taken up at the same level as the wild type Schu S4. Furthermore, the *F*. *tularensis* Schu S4 *waaY*::TrgTn strain displays a similar growth phenotype in human AT-II cells as that observed in human MDMs, in that the strains grow well for ~16 hours or so before growth levels off. The mechanism for why this mutation results in this cell growth phenotype is unknown and is under active investigation.

### Transmission electron microscopy of early *Francisella* interactions with murine lung tissue

The interactions between AT-II cells and various *Francisella* strains suggest that these pathogenic bacteria have the ability to cross the epithelial barrier by the internalization of *Francisella* into an epithelial cell. Additionally, if LVS replicates only poorly in AT-II cells *in vivo* these interactions may help to explain why the respiratory virulence of LVS for mice is significantly reduced compared to the virulent Schu S4 strain. Although there has been some investigation into the role of AT-II cells *in vivo*, to our knowledge there has never been any TEM evidence confirming internalization *in vivo*. To better understand the early events that lead to establishment of pneumonic tularemia and to determine if *F*. *tularensis* can be internalized into AT-II cells *in vivo*, mice were infected intranasally with *F*. *tularensis* Schu S4, Schu S4 *waaY*::TrgTn, LVS or PBS (negative control). In addition, to the intracellular growth defects observed in the Schu S4 *waaY*::TrgTn mutant, this strain displays decreased virulence in mice, and mice immunized with Schu S4 *waaY*::TrgTn demonstrated some protection against a wild type Schu S4 challenge [24, 35]. Furthermore, through observation of the mice and histopathology, the cause of death is different between Schu S4 and the *waaY* mutant, (multiple organ failure and suffocation due to fluid filled lungs, respectively) [35, 36]. These data suggest that the Schu S4 *waaY*::TrgTn strain induces a host response that is distinct from that caused by wild type organisms. At 16 or 24 hours post-infection, mice were euthanized and the lungs were perfused and processed for TEM imaging. A MOI of 100 bacteria per alveolus was used to increase the probability of detecting organisms within the lung tissues.

**Fig 4:**
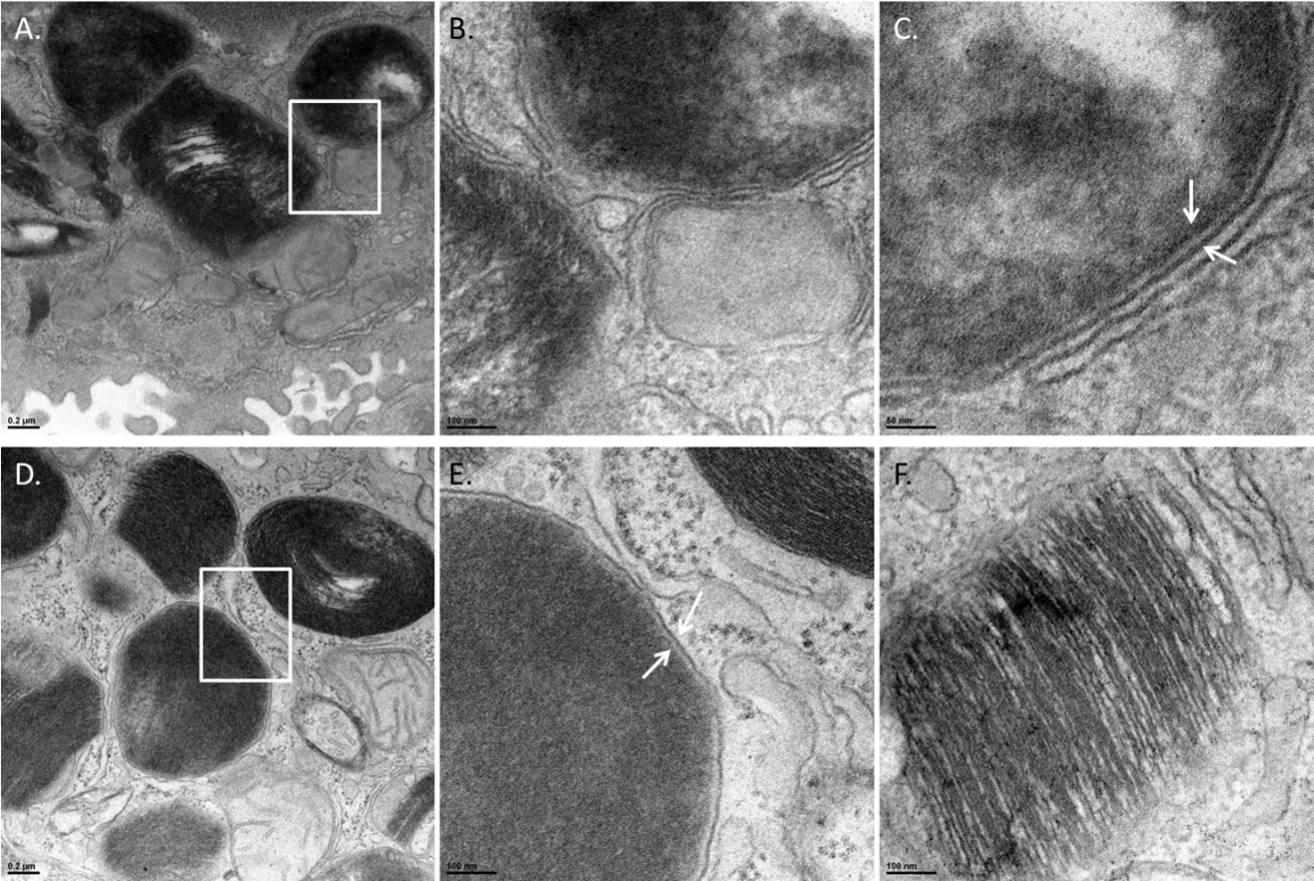
Identification of *F*. *tularensis* in AT-II cells. TEM images of infected mouse lungs at 24 hours post infection. To differentiate between lamellar bodies and bacteria in AT-II cells, infected cells were observed under high magnification. Lamellar bodies were classified as dark stratified stained organelles whereas; bacteria were identified by smooth dark staining that lacked stratification. Panel A—Cell containing a *F*. *tularensis* organism magnified 8,000-fold. White rectangle marks area of interest that is shown in panels B and C. Panel B is the area of the white rectangle in Panel A magnified 25,000-fold. A bacterium and the membrane surrounding the organism are shown. Panel C—The white arrows are pointing to the double membrane (outer and inner membrane) of *F*. *tularensis*. (Panel D) An intracellular organism magnified 8,000-fold. The white rectangle marks an area of interest shown in Panel E. Panel E is the area of the white rectangle in Panel D magnified 25,000-fold. Panel F—25,000-fold magnification of a lamellar body demonstrating the stratified staining from the packaged surfactant.

Organisms of each strain were observed in lung cells at either 16 or 24 hours post-infection, but none were observed in the lungs of mice inoculated with PBS as a control. Due to the staining and TEM imaging, some of the images taken at low magnification had dark staining that often made distinguishing *Francisella* from lamellar bodies (organelle that packages surfactant) difficult. However, upon higher magnification there were stark differences observed between the bacterium and lamellar bodies. *F*. *tularensis* was differentiated from lamellar bodies by dark, often smooth or bubbly staining (likely the chromosomal DNA) that was spread throughout the organism and the bacteria clearly lacked the stratified staining characteristic of the lamellar granules (Fig 4). Lamellar granules are organelles unique to AT-II cells that package and secrete surfactant and are visible in TEM imaging by their stratified staining that alternates between dark and white linear bands created from the tight packaging of surfactant membranes [11]. Furthermore, bacteria were also characterized by identification of a double membrane (Fig 4C). Using these characteristics, we were able to reliably identify bacteria of each strain in our TEM images.

**Fig 5:**
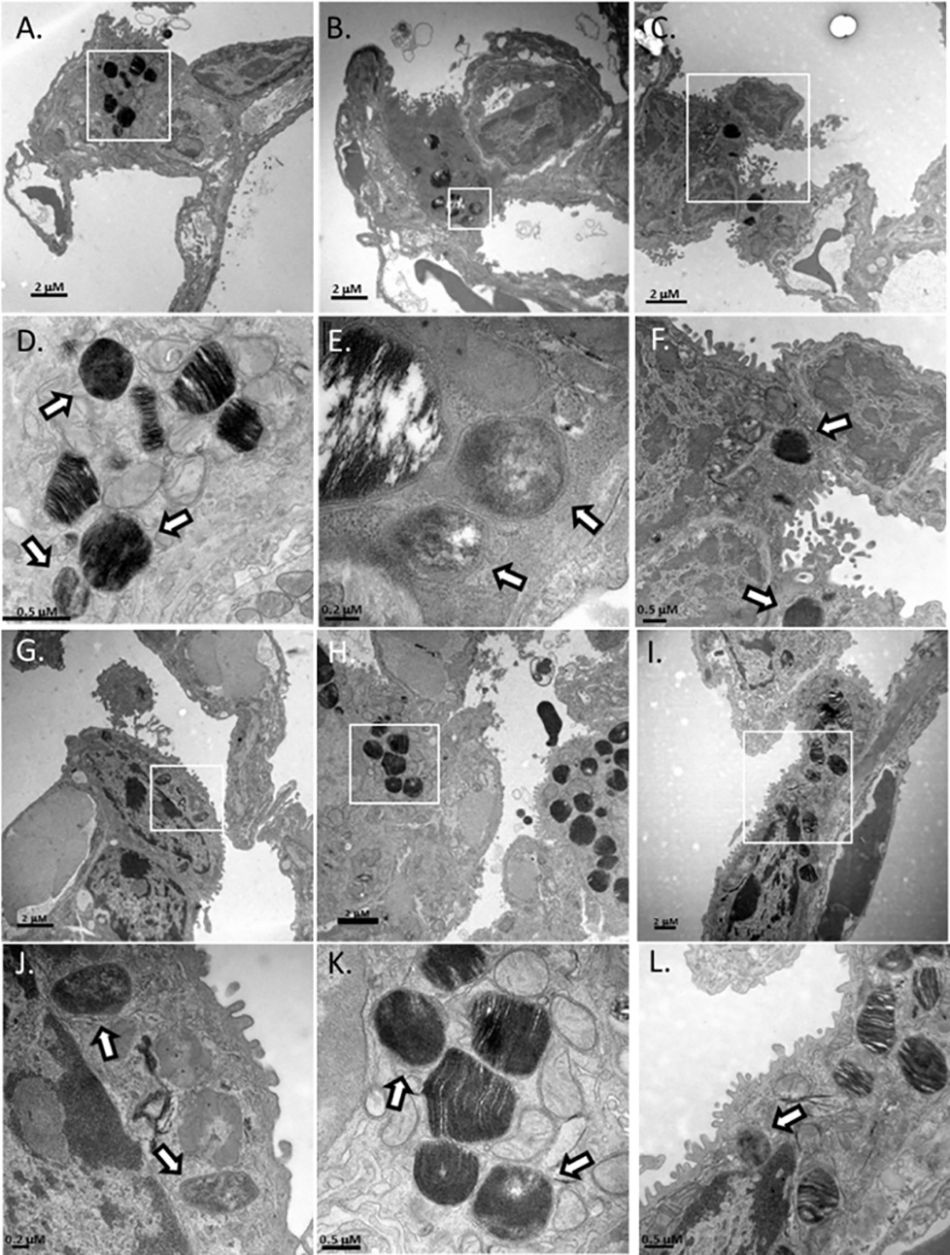
Electron microscopy images of *Francisella tularensis* strains within murine alveolar type II epithelial cells. TEM images of infected mouse lungs at 24 hours post-infection. Mice were infected intranasally with either *F*. *tularensis* Schu S4 (A-F), *F*. *tularensis* Schu S4 *waaY*::TrgTn (G-K), and *F*. *tularensis* LVS (I,L). Each image shows an AT-II cell containing organisms. The AT-II cell can be identified by the presence of microvilli at the cell-air interface and by the presence of lamellar granules. It is also worth noting that each infected AT-II cell was immediately adjacent to a pulmonary capillary. The area containing the internalized bacteria is within the white rectangle which is shown at higher magnification immediately beneath the corresponding image. The arrows identify the bacteria in each field.

In examining infected lung tissue we occasionally, although rarely, observed an alveolar macrophage containing an internalized organism that was within the airspace of an alveolus (5 cells total across all samples, data not shown). The most common cell type observed to contain *Francisella* were AT-II epithelial cells (Fig 5). AT-II cells were identified as the cells that produced microvilli at the cell::air interface and contained lamellar granules. Multiple AT-II cells were observed that contained 1–3 bacteria from lungs infected with each of the three *Francisella* strains (Fig 5). It was also interesting to note that the AT-II cells typically bordered pulmonary capillaries, highlighting that once the organisms entered an AT-II cell they could gain access to the bloodstream with little impediment. Furthermore, while there might be small differences in quantitative uptake of the different *Francisella* strains into the AT-II cells, no differences could be determined using TEM.

### Observable cell death in AT-II cells with intracellular *F*. *tularensis*

By 16 and 24 hpi *Francisella* had internalized into murine AT-II cells *in vivo*, but at these time points it was not possible to determine the fate of the *Francisella*-infected cells (conducive for bacterial replication or cell death). As this is an *in vivo* infection it is possible that 24 hpi is not sufficient time for the bacteria get into the alveolar space, internalize into AT-II cells, and replicate. To better understand the outcome of infected airway epithelial cells, we infected mice intranasally with Schu S4, Schu S4 *waaY*::TrgTn, or LVS. Mice were sacrificed at 32 or 48 hpi and lungs were harvested. Lung tissues were processed as described previously and TEM was performed.

**Fig 6:**
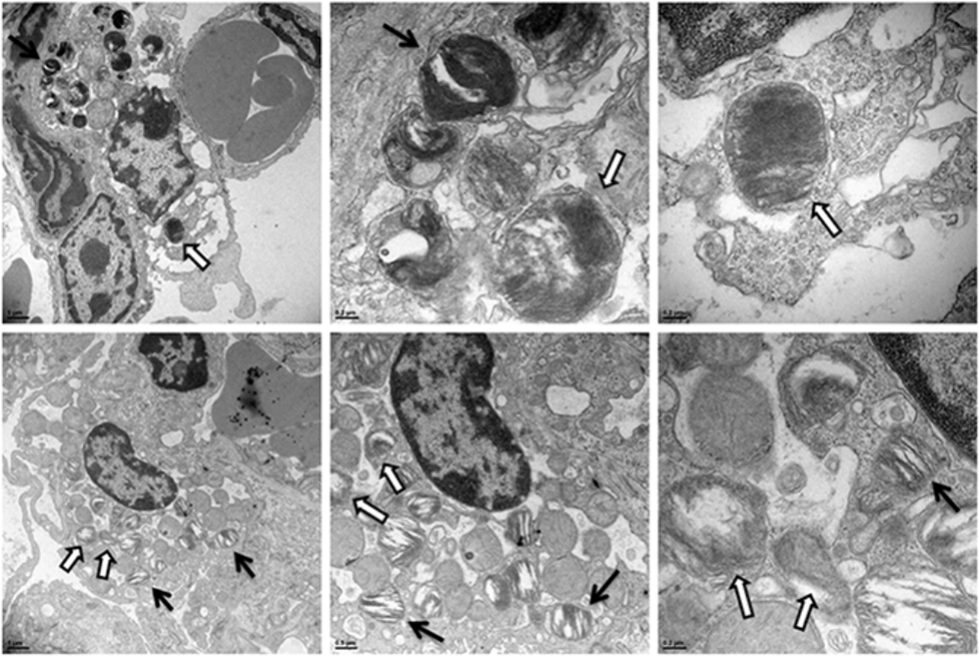
TEM of *F*. *tularensis* in dying AT-II cells *in vivo*. TEM images of infected mouse lungs at both 32 and 48 hpi infected with Schu S4. (A-C) AT-II cells containing *Francisella* going through cell death characterized by lack of electron density of the cytosol and swollen mitochondria. (D-F) A separate AT-II cell undergoing cell death infected with *Francisella*. The open arrows identify the bacteria in each field and the solid black arrows indicate lamellar bodies in the cells.

Similar to infected lungs at 16 and 24 hpi, the majority of the cells containing internalized *Francisella* were AT-II cells. However, unlike cells with intracellular *F*. *tularensis* at 16 and 24 hpi, several infected AT-II cells had visible changes suggesting that the cells were dying (Fig 6). Infected AT-II cells were observed to have loss of cytoplasmic density, swelling of mitochondria, and disrupted mitochondrial cristae structure, which have previously been reported in dying cells observed by TEM imaging [37–39]. These dying cells were observed in all strains tested and no significant differences between strains were observed by TEM imaging. In addition, cells typically had only a few organisms present indicating that there was no significant replication of the bacteria within the AT-II cells. However, as TEM imaging is with sections that are 70nm thick, the lack of observable replication may be due to limitations from TEM imaging.

### The *F*. *tularensis waaY*::TrgTn mutant induces substantial airway pathology that is not induced by wild type *F*. *tularensis* Schu S4

**Fig 7:**
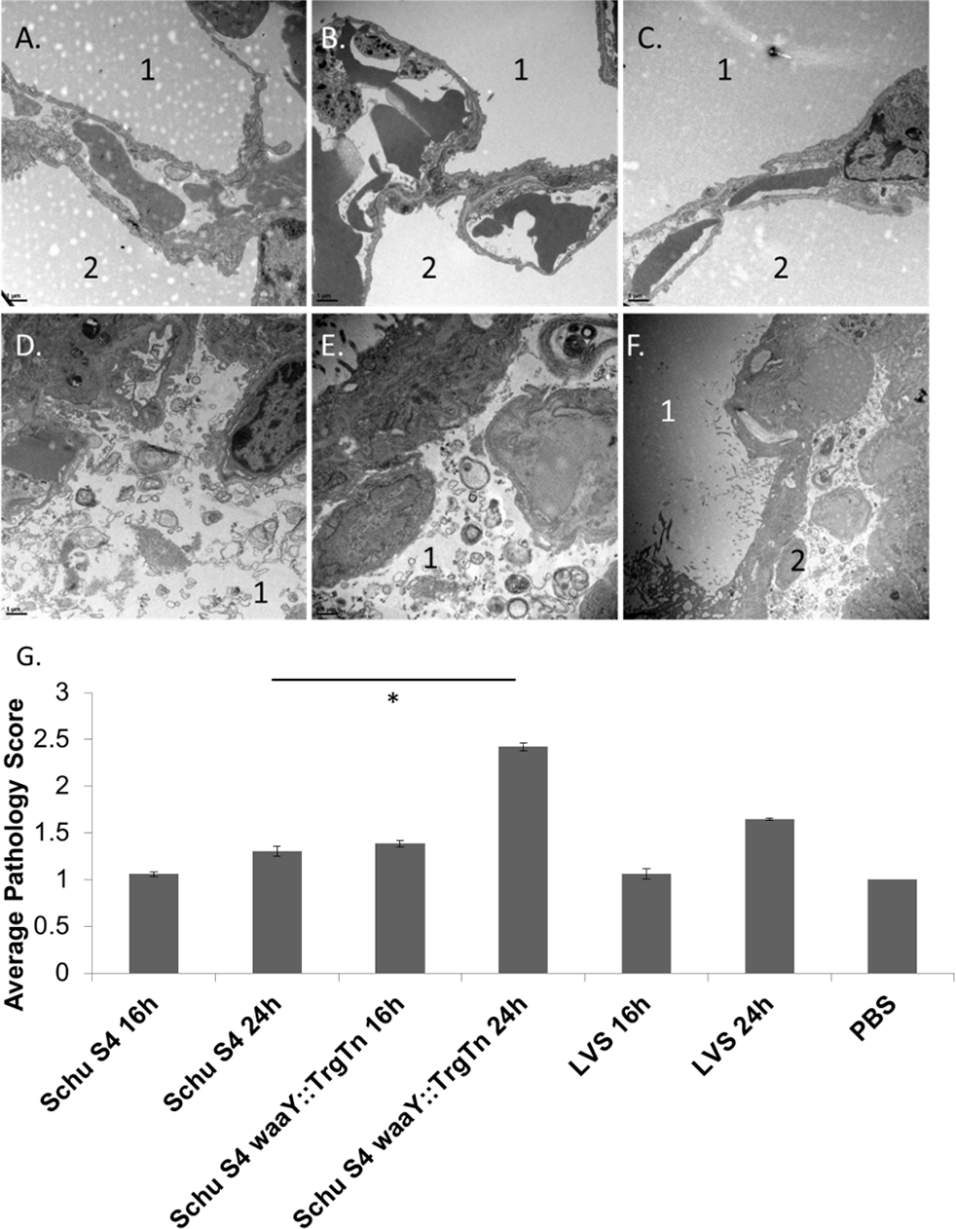
Schu S4 *waaY*::TrgTn increases cellular debris within the alveoli. (A-C). TEM imaging of 24 hours demonstrating clean airspace with the alveoli in lung tissue that is infected with either PBS (A), Schu S4 (B), or LVS (C) and airspace that is representative of lung tissue infected with Schu S4 *waaY*::TrgTn (D-F). B. Graphical representation of scoring of the airspace for cellular debris. There was an increase in cellular debris in lungs infected with Schu S4 *waaY*:TrgTn compared to PBS control (*P* < 0.001). Averages were calculated from analysis of greater than 50 scored airspaces from 2 separate lung sections per strain and time point.

While we could not detect quantifiable differences in bacterial uptake into AT-II cells *in vivo*, there were strain-specific differences in the pathology that was observed in the airway spaces following infection. At 16 and 24 hours post-infection, lungs infected with either PBS, Schu S4, or LVS were essentially clear of debris within the airspace observed (Fig 7A, 7B and 7C). However, mice infected with Schu S4 *waaY*::TrgTn at 16 hours post-infection had significant pathology in the alveoli, as evidenced by large amounts of debris in the alveolar airspaces that contained both surfactant and cellular components. By 24 hours post infection with this mutant, a substantial portion of the airspace was observed to contain this cellular debris (Fig 7D, 7E and 7F).

To more accurately evaluate the damage induced in the lungs by the *F*. *tularensis waaY*::TrgTn mutant, semi-quantitation of airspace debris was scored using TEM images [40]. Individual alveolar airspaces, defined by the border of the alveolar epithelium, were evaluated for the percent of the airspace containing cellular debris and were given a score of 1–4, with 1 having less than 25% of the airspace filled with debris and 4 being greater than 75% of the airspace filled with debris. Approximately, 50 TEM fields were evaluated per strain and time point. In the PBS control mice, there was little to no cellular debris observed at 16 or 24 hours post infection (Fig 7G). Additionally, there was no significant cellular debris observed at 16 hours post-infection for *F*. *tularensis* Schu S4 or *F*. *tularensis* LVS compared to the control PBS infected mice (Fig 7G). At 24 hours post-infection, both Schu S4 and LVS had some observable cellular debris that increased the average airspace damage score from 1 to 1.3 and 1.6, respectively (Fig 7G). *F*. *tularensis waaY*::TrgTn had observable cellular debris with an average score of 1.3 at 16 hours post-infection, which was comparable to wild type Schu S4 at 24 hours post-infection. By 24 hours post-infection, there was significantly more cellular debris induced by the Schu S4 *waaY*::TrgTn strain than the PBS control (P < 0.001), with an average score of 2.4, meaning that the average airspace had 50–75% filled with cellular debris (Fig 7G). Furthermore, of the airspaces scored from lungs infected with Schu S4 *waaY*::TrgTn at 24 hpi, 38% of samples scored had over half of the airspace filled with cellular debris and 28% of the airspaces scored had over three-fourth of the airspace filled. Comparatively, wild type Schu S4 only had 3% of all scored airspaces that contained over half of the airspace filled and no airspace was observed in Schu S4 infected lungs to have greater than three-fourth of the airspace filled with cellular debris. The mechanism by which the *waaY*::TrgTn mutant induces the accumulation of debris in the lung airspaces of mice is unknown but is likely directly related to the other properties of this mutant, such as the induction of early cell death of MDMs and attenuation *in vivo* [24, 35].

## Discussion

*F*. *tularensis* is a Tier 1 Select Agent pathogen that is capable of causing lethal human respiratory infection with a very low inoculum. In order to cause disease, organisms in the airway must breach the epithelium and pass through the endothelium to enter the bloodstream, which allows the organisms to disseminate from the lung to the liver and spleen. While significant effort has been expended to study the interactions of these pathogens with various host cells, the mechanisms by which *Francisella* passes through the respiratory epithelia are still poorly understood. Much of the research has focused on the interactions of *Francisella* with macrophages *in vitro*, but it is not clear how the interactions of *Francisella* with alveolar macrophages would facilitate the passage of the bacteria through the respiratory epithelium *in vivo*. Alveolar macrophages are found within the airspaces, and are associated with the apical surface of the airway epithelium. These cells have high phagocytic activity and protect the lung epithelium by engulfing particles or organisms from the inhaled air. Following engulfment of particles, the cells detach from the airway epithelium and are typically expelled from the airway via the mucociliary escalator [8]. Since the established role for alveolar macrophages is to engulf particles and organisms from the airspaces and remove them from the host via the mucociliary escalator, it is difficult to understand how *Francisella* uses alveolar macrophages to penetrate into lung tissue although, of course, it is still possible that *Francisella* uses these cells to breach the lung epithelium.

Type II pneumocytes, or AT-II cells, are an integral part of the alveolar epithelium, comprising 15% of all lung cells [9] and have been implicated by several groups as a lung cell type in which *Francisella* can grow [19, 21, 22]. AT-II cells have a variety of biological functions including production of surfactant that is important for biophysical stabilization of the alveolus [41], maintenance of alveolar fluid balance [42], differentiation into AT-I cells which are important for structural integrity of the alveolus [43], and involvement with activating components of both innate and adaptive immunity [17]. Specifically, these cells have been shown to constitutively express several toll-like receptors, along with NLR proteins, which are involved in the production of cytokines that recruit and interact with dendritic cells, T cells, and B cells [23, 44, 45].

In an effort to establish a cell culture model that closely mimics *Francisella* interactions with lung epithelium during infection of a host, we acquired a newly available human AT-II cell line to study the entry and growth of *Francisella* in a relevant epithelial cell line. The initial uptake of *Francisella* species (Schu S4, Schu S4 *waaY*::TrgTn, and LVS) into these cells was comparable to that observed for other tissue culture non-phagocytic cell lines such as HEp-2, HeLa, HEK-293, COS-7 and A549 cells, as observed by us and other groups [19, 21, 22]. Several research groups have studied various properties of *F*. *tularensis* strains in the human lung epithelial cell line A549 [46–49]. One group compared the ability of *F*. *tularensis* Schu S4, a Schu S4 Δ*purMCD* mutant, *F*. *tularensis* LVS and a LVS Δ*purMCD* mutant to grow in both human macrophages and A549 cells. While the work was focused on evaluating the importance of the Δ*purMCD* genes as a virulence factor, the results demonstrate no discernible growth differences between *F*. *tularensis* Schu S4 and *F*. *tularensis* LVS in A549 human lung epithelial cells [46]. Others demonstrated that LVS grows well in A549 cells [48, 50], but did not compare the growth of LVS to a virulent *F*. *tularensis* strain. Another group has attempted to develop a more relevant cell model for studying *Francisella* interactions by growing A549 cells in rotating-wall vessels (RWV) to stimulate *in vivo*-like phenotypes [49]. However, their work did not detect any significant growth differences between *F*. *tularensis* Schu S4 and *F*. *tularensis* LVS in A549 cells, although the RWV growth conditions did seem to stimulate cells to express cell polarity markers and other proteins indicative of cells growing *in vivo*.

In this work, we have observed a significant difference in the ability of *F*. *tularensis* Schu S4 and *F*. *tularensis* LVS to grow within immortalized primary human AT-II cells after uptake. Virulent Schu S4 grew to high numbers in the cytosol of these cells (1600-fold growth was observed from 4 hours to 24 hours) while the LVS strain exhibited minimal growth in the human AT-II cells (~ 2- to 12-fold growth). We believe that this is a significant finding since to our knowledge these human AT-II cells are the first cells where such a significant difference in growth exists between virulent *F*. *tularensis* and the vaccine (attenuated virulence) strain *F*. *tularensis* LVS. This correlation between virulence and growth may provide an important opportunity to gain insights into the genetic reasons for the attenuation of the LVS strain which may then provide the opportunity to engineer a better vaccine strain. However, as we have previously reported for Schu S4 growth in MDMs (reference), we saw little evidence for cell death in the primary AT-II despite the presence of such large numbers of bacteria in the cytosol of the host cell. Thus, while we believe that these immortalized primary human AT-II cells are a better model system for studying *Francisella*-nonphagocytic cell interactions than long established tissue culture lines, they still do not replicate all aspects that are observed of *in vivo* infections. Particularly, we observed significant cell perturbations in murine AT-II cells infected with low numbers of Schu S4 organisms (1–3 organisms visible in a given EM section) which was not observed in the cultured human AT-II cells that contained hundreds to thousands of replicating Schu S4 organisms.

The *F*. *tularensis waaY*::TrgTn strain also has an attenuated virulence phenotype in the human AT-II cells. Since previous work from our lab has demonstrated that this strain has a significant growth defect in human MDMs, it is possible that this strain is unable to replicate to high numbers in airway epithelial cells for the same reason. Work is currently underway to characterize the mechanism that leads to cessation of bacterial growth and cell death in both cell types. An understanding of this mechanism may help clarify how host cells combat intracellular bacterial pathogens.

Additionally, we performed experiments to determine what host cells are the early targets of *F*. *tularensis* as they establish infection of the mouse lung. Murine lung tissues from mice infected with *F*. *tularensis* were examined by transmission electron microscopy to identify the cell types that contained intracellular *F*. *tularensis*. We spent considerable effort looking for organisms within alveolar macrophages but observed very few *Francisella* organisms within these cells (~5 infected alveolar macrophages in all samples imaged). It is possible that there were more alveolar macrophages that had engulfed organisms than were detected during these experiments since this is the primary function of the cells. However, as alveolar macrophages typically detach from the epithelium after phagocytosis, it seems likely that alveolar macrophages that had engulfed *Francisella* organisms may have been washed out of the mouse lungs by perfusion. Alternatively, these alveolar macrophages could have been removed by the mucociliary escalator in the mouse lungs as part of the normal defense mechanisms before lungs were extracted and fixed. In either case, the absence of alveolar macrophages containing *Francisella* organisms in the infected lung samples provides support that interactions with other cell types are important for penetration into the lung tissue.

Careful examination of the infected mouse lung tissue revealed that organisms were consistently observed within AT-II cells by 16–24 hours post-infection for each of the three strains used in our experiments (Schu S4, Schu S4 *waaY*::TrgTn or LVS). It is not a trivial exercise to identify intracellular *Francisella* organisms within the AT-II cells of the alveoli by TEM, as there are an estimated 1 x 10^6^ alveoli per mouse lung and many AT-II cells per alveoli. In addition, sectioning of the lung tissue captures only a small percentage of the cellular material per alveoli for viewing, which also decreases the probability of finding evidence of intracellular bacteria. Since we were able to find bacteria within the AT-II consistently, despite these hurdles, our data extends previous studies suggesting that these cells are an important target cell in the alveolar epithelium. The TEM images highlight the path that organisms within the AT-II cells likely follow to cause systemic disease. Following entry into the AT-II cells, the bacteria are immediately adjacent to alveolar capillaries. As organisms pass through or escape from an AT-II cell they will have access to the bloodstream via passage through the endothelium of the immediately available alveolar capillary.

As later time points were examined, we consistently observed infected AT-II cells that were undergoing cellular stress and death. These cells had several characteristics of dying cells such as loss of cytoplasmic electron density, swelling of mitochondria, and disruption of mitochondrial structure. A possible explanation for this phenomenon could be that the high inoculum of organisms, which was used so that individual infection events could be discovered within the relatively large lung, was causing non-specific tissue destruction. However, this seems unlikely as we only observed AT-II cells, which contained internalized bacteria undergoing cell death and any tissue damage due to inoculum would likely cause damage to multiple cell types, including uninfected AT-II cells, which was not observed. In addition, it is well-established that cells with intracellular *F*. *tularensis* undergo cell death by 24–48 hpi as part of the pathogenesis of the organism. Unfortunately, we were not able to determine if there was replication within the AT-II cells by use of TEM imaging, as we were unable to examine specific cells over a sufficient period of time. Future efforts to address this issue would need to rely on other techniques, such as confocal microscopy, to determine if AT-II cells are permissive for replication of *F*. *tularensis in vivo* or if AT-II cells primarily function as a gateway through the epithelial barrier.

The inclusion of the *F*. *tularensis* Schu S4 *waaY*::TrgTn mutant in this work provided important contrasts to the interactions of wild type *F*. *tularensis* Schu S4 and LVS within lung tissue. This mutant strain induced significant levels of pathology in the mouse lungs in the form of debris (inflammation) that was present in the airspaces. Mouse lungs infected with Schu S4 or LVS were virtually free of inflammation at 16 hours post infection in comparison, as noted by the lack of debris-filled airspace. These data are not surprising because other work has demonstrated that *F*. *tularensis* fails to induce inflammation upon infection as it does not stimulate pattern recognition receptors and by inducing broad immunosuppression [51]. The *waaY* gene was identified as being important in the ability of *F*. *tularensis* Schu S4 to grow in human MDMs [24] and subsequent work revealed that the strain did not produce normal LPS or capsule. More recently, our group has shown that the strain is defective for mouse virulence and sublethal infection of mice with this strain provides protection against lethal doses of wild type Schu S4 [35]. This work reveals that this strain induces host cell responses in the lung in the early stages of infection that seem to significantly alter host recognition of the strain and the course of disease. Part of this host response is likely to be initiated by early death of the host cell, as our lab has previously shown occurs with this mutant strain [24]. An understanding of the mechanism by which this *Francisella waaY*::TrgTn strain induces host cell death is an ongoing interest in the lab since avoidance of detection by the host is a key virulence strategy of *Francisella* and may provide important clues to developing an effective therapy against this potential bioweapon. In contrast to the mutant strain, the lack of cellular debris in wild type infected lungs adds to the evidence that virulent wild type *Francisella* strains establish infection of a host using a combination of active pathogenic strategies (host cell entry, phagosome escape, cytosolic growth that allows systemic distribution) and stealth strategies (evasion of host detection by non-stimulatory LPS and suppression of inflammatory signals by unknown mechanisms) [52–54].

**Fig 8:**
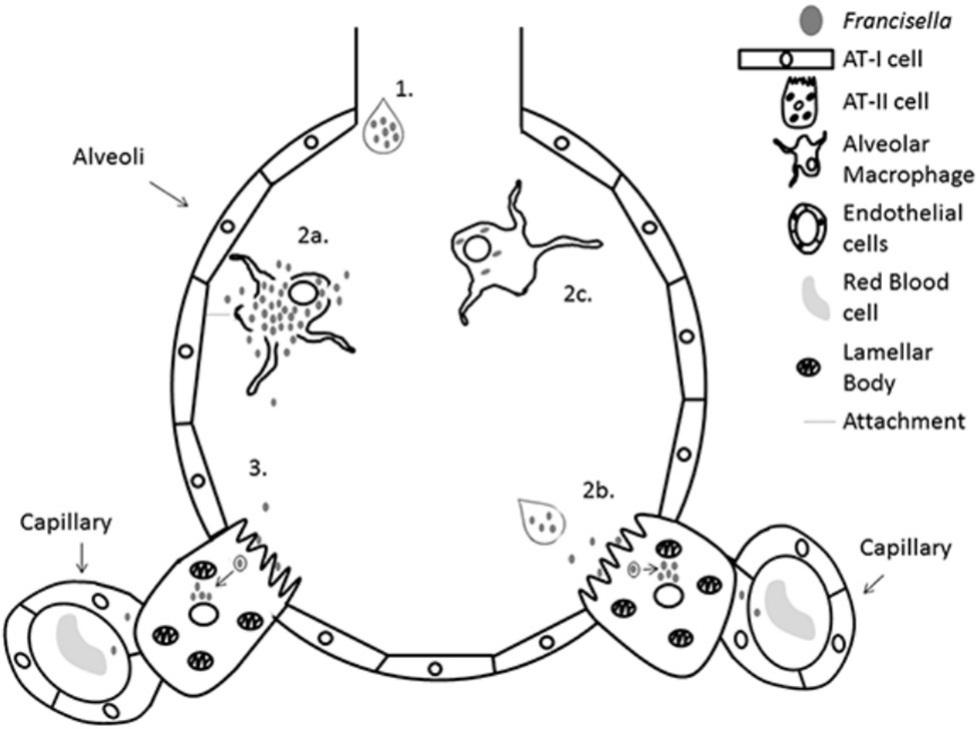
Model of early *Francisella* infection within the lung. (1) *Francisella* enters into the alveoli from an aerosolized infection where it either gets phagocytosed by alveolar macrophages (2a) or interacts with AT-II cells (2b). Upon uptake into alveolar macrophages there are at least two possible outcomes, either alveolar macrophages allow bacterial growth and release into the airspace (2a) or alveolar macrophages detach and are removed from the alveoli by the mucociliary escalator (2c). Growth and release from the alveolar macrophages allows reinfection with surrounding tissue including AT-II cells (3). Internalization with AT-II cells acts as a mechanism to get past the epithelial barrier and allows for interaction with endothelial cells and eventually dissemination to the liver and spleen.

Taken together, the TEM data and the *in vitro* cell infection data have allowed us to develop a model of how *F*. *tularensis* establishes lung infection (Fig 8). Inhaled *F*. *tularensis* organisms are carried to the small alveolar sacs and interact with the cell populations present in the alveoli: alveolar macrophages and AT-II cells. If *Francisella* first interacts with an alveolar macrophage there are at least two possible outcomes: 1-The macrophage engulfs an organism, detaches from the epithelium, and is removed from the alveolus via the mucociliary escalator or 2- an organism is phagocytosed and the infected alveolar macrophage remains in the alveolar space, where *Francisella* replicates to large numbers, escapes from the cell and re-infects adjacent cells in the epithelium. This re-infection may allow contact of *Francisella* with AT-II cells where the bacteria could pass through the epithelial barrier by entering and growing within AT-II cells. It is also possible that upon inhalation, *Francisella* first interacts with an AT-II cell. This interaction would also allow for the passage of the bacteria through the epithelial barrier via the AT-II cell. Once invaded, AT-II cells will lyse from growth of intracytoplasmic bacteria, releasing *Francisella* organisms into the environment. The newly released bacteria would have access to the endothelial vessels and could quickly disseminate to the liver and spleen through the blood. This strategy suggests that most *F*. *tularensis* organisms would enter into the bloodstream as extracellular organisms. In support of this model, a study by Forestal *et al*. demonstrated that 75% of Schu S4 organisms in the bloodstream were extracellular [55]. Future experiments will help to clearly define the individual steps in the infection process which should help to identify targets to better treat tularemia and/or to understand the host-pathogen interactions that may help to develop a live attenuated vaccine.
